# Hospitals and the War

**Published:** 1915-01-09

**Authors:** 


### HOSPITALS AND THE WAR.

**(From our Special Correspondents.)**

### MORE WOUNDED ARRIVE AT CAMBRIDGE.

On Christmas Eve a hospital train steamed into Cambridge Station, bringing another 150 wounded soldiers from the seat of war. They were very expeditiously removed in ambulances and motors, which were in readiness, to the 1st Eastern General Hospital.

Yet another batch of wounded arrived on Christmas Day in a hospital train, numbering about 100, and nearly the whole of them cot cases. Such an exceptionally large number of cot cases arriving in one day, needless to say, taxed the resources of the Cambridge Branch of the British Red Cross Society to the utmost, particularly the supply of ambulances.

On Sunday night, January 3, 1915, at a very late hour, fourteen wounded officers from the Front were brought in an ambulance coach attached to the mail train from Liverpool Street to Cambridge, where they arrived about 11.26. The ambulance coach was detached from the mail on its arrival at Cambridge and shunted on to the Great Eastern line near the waiting room.

The 7th Men's Voluntary Aid Detachment, commanded by Dr. Wood, were in waiting at the station, and they removed the seven stretcher cases from the ambulance coach to the cars in readiness. The other seven officers were able to walk to the cars. Both they and the stretcher cases were in a short time conveyed to the Research Hospital, Hills Road, Cambridge.

### CARDIFF.

There were many cot cases among the 123 soldiers who arrived in Cardiff on the evening of December 28, this contingent numbering a total of 303 received within thirty-six hours. The weather was very bad, and the draughty platform was a source of anxiety to the officers. Colonel East, D.S.O., was present, and Major Maclean was in charge of the detraining. Captain Nicholson was also in attendance, and Mr. W. Harry was in charge of the Red Cross men, who numbered nearly 100.

One of the men who had been hit by shrapnel was wearing the cap through which the missile had torn its way. A Royal Welsh Fusilier, who had been at the Front since August 9, said that he last saw fighting at Ypres, where he was wounded in the face. He stated that he had seen men in the trenches pulling at their boots for over twenty minutes, in an effort to get out of the mud, and finally going out without their boots, which were still there! Another soldier said that he had been in the trenches barefooted, and spoke of the terrible scenes which he witnessed when the trenches were flooded. The party had a quick run in France, but the sea journey was very rough. They eulogised the splendid way in which they had been conveyed from the Front, despite exceptional difficulties and dangers. Fifty-five cases went to Albany Road, fifty to Splott Road, fifteen to Howard Gardens, and three to King Edward VII.'s Hospital.

### GLAMORGAN AND MONMOUTHSHIRE HOSPITAL.

The staff of the Glamorgan and Monmouthshire Hospital, under Dr. H. G. Cook, of Cardiff, have arrived at their destination and taken up their quarters at the hotel which had been placed at their disposal for use as a hospital at Berck Plage. The district is regarded as a very suitable hospital centre owing to its fine sea air, but ambulances are urgently wanted owing to the bad railway accommodation. Some £4,000 has been obtained, but £6,000 at least is required.

### NETLEY: CHRISTMAS AT THE WELSH WAR HOSPITAL.

The ninety-three soldiers from the Front in the Welsh War Hospital at Netley were given a most enjoyable time. The wards presented a very bright appearance with their decorations and blue and white colourings, and the men received not a few presents, including a charming memento from Sir William James Thomas in the shape of a silver match-box, with the words "Welsh Hospital, 1914," on it, and accompanied by a card wishing each a speedy recovery. There was Divine service in one of the wards, a splendid Christmas dinner, and afternoon concerts. On Christmas Eve the musical members of the staff formed a carol party, and visited the whole of the wards and the quarters of the commanding officer. All the staff received a present from Sir W. J. Thomas, and a Christmas dinner for the officers and nursing staff in the General Store, specially decorated for the occasion, concluded a very successful if strenuous day.

### SHEFFIELD: 3rd NORTHERN GENERAL HOSPITAL.

The Right Hon. Sir Frederick Milner, Bart., on behalf of their Majesties the King and Queen, recently visited the 3rd Northern General Hospital, Sheffield, and in a personal conversation with Colonel Dr. Connell, who has charge of the hospital, he expressed himself as being delighted with everything he saw. Sir Frederick visited the various wards and gave the wounded "Tommies" a cheering message from the King and Queen. Furthermore, each British soldier who had fought in France or Belgium and each Belgian soldier in the hospital was presented with a neat box containing chocolates or cigarettes as desired.

Many of the people of Sheffield have been most kind and generous in the help they have rendered Colonel Dr. Connell by taking one or more of the wounded into their private houses when the men have become sufficiently convalescent. Over sixty Belgian wounded have been taken care of in private houses in Sheffield in this way. Then in the surrounding district many county people have been only too anxious to do whatever they could for the wounded, and the numbers of men they have taken has proved an immense help to Colonel Dr. Connell.

The Duke of Rutland has kindly placed Longshaw Lodge, Derbyshire, at the disposal of Colonel Dr. Connell for use as a convalescent auxiliary hospital. There will be fifteen beds, and Mrs. Hutton, of Grindleford, will have charge.

